# Cognitive function among military veterans with STEM occupations

**DOI:** 10.1186/s40779-023-00491-7

**Published:** 2023-11-20

**Authors:** Justin T. McDaniel, Kevin N. Hascup, Erin R. Hascup, Ugochukwu G. Ezigbo, Amanda M. Weidhuner, Harvey Henson, David L. Albright

**Affiliations:** 1 Dale and Deborah Smith Center for Alzheimer’s Research and trEatment (CARE), Southern Illinois University School of Medicine, Springfield, IL 62794 USA; 2 STEM Education Research Center, Southern Illinois University, Carbondale, IL 62901 USA; 3 Department of Political Science, The University of Alabama, Tuscaloosa, AL 35487 USA

**Keywords:** Alzheimer’s disease, Veterans, Science, technology, engineering, and mathematics (STEM), Animal fluency test, Dementia

Dear Editor,

There is limited research on the relationship between science, technology, engineering, and mathematics (STEM) occupational history and cognitive function in later life, especially among military veterans, who may be at greater risk for later-life cognitive decline. This study examines a nationally representative sample of military veterans to address this gap in knowledge.

We obtained data for this cross-sectional study from the 2011–2014 waves of the National Health and Nutrition Examination Survey [1] on veterans (*n* = 464) and civilians (*n* = 2093). Cognitive function was assessed using three tests: the immediate and delayed memory word recall test [2], the animal fluency test [3], and the digit symbol substitution test [4]. We calculated an index of word loss from the immediate to delayed 10-item word recall test (immediate score – delayed score), such that higher scores on the index indicated worse cognitive function. In the other two tests, higher scores were indicative of better cognitive performance.

The primary independent variable is STEM occupational history, operationalized through the question: “Thinking of all the paid jobs or businesses you ever had, what kind of work were you doing the longest?” We grouped occupations into two categories: STEM or non-STEM. We also include healthcare practitioners—but not medical support staff—in the STEM category. We controlled for age, sex, race, educational attainment, diabetes status, healthy diet, minutes of sedentary behaviour—except for sleeping—on a typical day, depression, traumatic brain injury, illicit drug use, and benzodiazepine use. We estimated multivariable linear regression models in which the three cognitive test scores were regressed on STEM involvement, veteran status, all covariates, and an interaction term for STEM occupation and veteran status using the following formula:

$$\begin{aligned} Cognitive\; Function & = a + b\left( {age} \right) + b\left( {race} \right) + b\left( {sex} \right) + b\left( {HS \;diploma} \right) \\ & \quad + b\left( {some\;college} \right) + b\left( {bachelor^{\prime}s\; degree\; o\;r more} \right) \\ & \quad + b\left( {diabetes} \right) + b\left( {healthy \;diet} \right) + b\left( {sedentary\; minutes} \right) \\ & \quad + b\left( {TBI} \right) + b\left( {depression} \right) + b\left( {illicit drug use} \right) + b\left( {benzodiazepine\; use} \right) \\ & \quad + b\left( {STEM} \right) + b\left( {veteran} \right) + b\left( {STEM \;x \;veteran} \right) + e \\ \end{aligned}$$

Participants (median age = 62.6) were primarily female (53.2%) and non-white (61.9%). Regarding the health status of the sample, results showed that 41.6% of the sample had diabetes, 16.8% had a traumatic brain injury, 19.1% had depression symptoms, 1.0% used benzodiazepines, and 16.7% had used illicit drugs. Full details about the study participants are presented in Additional file 1: Table S1. STEM occupational involvement was similar among veterans (6.5%) and civilians (7.6%). Veterans, when compared to civilians, scored higher on the animal fluency test (18.4 vs. 17.1) and digit symbol test (49.6 vs. 48.3), but exhibited greater forgetfulness on the word recall test (1.9 vs. 1.3).

Our multivariable linear regression models (Additional file 1: Table S2) revealed that the interaction term for STEM involvement and veteran status was associated with cognitive function as measured by the animal fluency test (b = 2.14, *P* = 0.03), but not the word recall test (b = − 0.11, *P* = 0.69) nor the digit symbol test (b = − 3.71, *P* = 0.12). For those with STEM involvement, mean score differences between veterans (M = 21.35) and civilians (M = 19.56) on the animal fluency test were also clinically meaningful (*Cohen’s d* = 0.76). In all models, the model predicted means on each test by veteran status and STEM involvement are shown in Fig. 1. Since there were only 3 identified female veterans, we also estimated a model delimited to the male veteran sub-group—results of which are presented in Additional file 1: Table S3. This delimited model showed that STEM involvement in male veterans was associated with cognitive function, as measured by the animal fluency test and the word recall test.

**Fig. 1:**
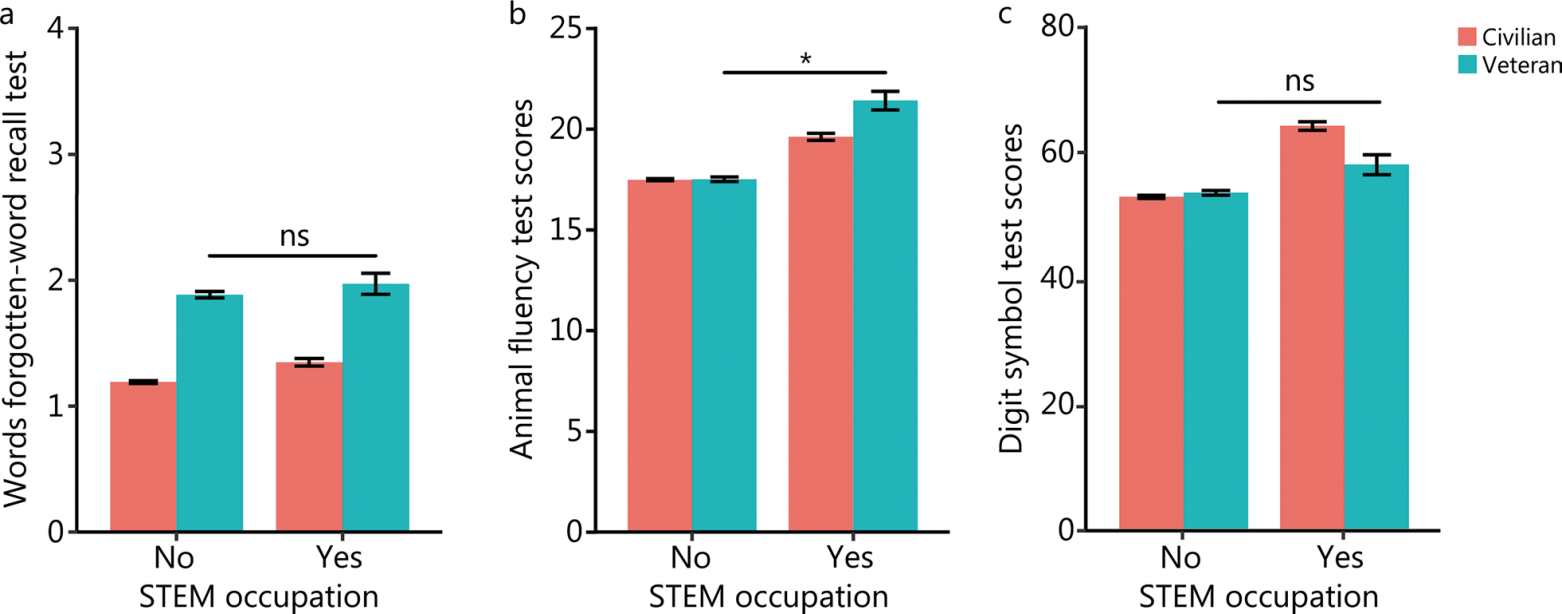
The predicted mean scores from the model in Additional file 1: Table S2. **a** Word recall test (i.e., forgetfulness from time 1 to time 2). **b** Animal fluency test. **c** Digit symbol test based on STEM occupational history and veteran status. Standard error bars are included. *P < 0.05 for the STEM occupational history and veteran status interaction term. Data were retrieved from NHANES waves between 2011 and 2014. ns non-significance

We used nationally representative data to examine the association between STEM occupational history and cognitive function among civilians and veterans. Because limited research exists in this area, particularly for veterans, this study provides basic preliminary evidence about said relationship. Although our study was limited by the cross-sectional study design (i.e., we could not make causal inferences about cognitive decline), results suggest that cognitive function may be better in veterans who have had a STEM occupation than those who have not. Future studies should explore veteran cognitive function by rurality, as previous reports have shown that rural-dwelling veterans are less likely to be involved in a STEM career [5].

### Supplementary Information

**Additional file 1: Table S1** Univariate descriptive statistics for all variables in the study stratified by military service status. **Table S2** Multiple linear regression results for the relationship between STEM occupational history, veteran status, and cognitive function test scores (*n* = 2557) [b (SE)]. **Table S3** Multiple linear regression results for the relationship between STEM occupational history and cognitive function test scores in military veterans who reported male sex (*n* = 461) [b (SE)].

## Data Availability

Data are available on the NHANES website (https://www.cdc.gov/nchs/nhanes/index.htm).

## Abbreviation

STEM: Science, technology, engineering, and mathematics
