# Supplementary material for: Cognitive function among military veterans with STEM occupations
Source: Mil Med Res. 2023 Nov 20;10:55. doi: 10.1186/s40779-023-00491-7 (PMC10658838; doi:10.1186/s40779-023-00491-7)
Supplement: Supplementary file 1 — Additional file 1: Table S1 Univariate descriptive statistics for all variables in the study stratified by military service status. Table S2 Multiple linear regression results for the relationship between STEM occupational history, veteran status, and cognitive function test scores (n = 2557) [b (SE)]. Table S3 Multiple linear regression results for the relationship between STEM occupational history and cognitive function test scores in military veterans who reported male sex (n = 461) [b (SE)]. [file 40779_2023_491_MOESM1_ESM.pdf]

Table S1 Univariate descriptive statistics for all variables in the study stratified by military service status

**Variable**	**Civilian (*n* = 2093)**	**Veteran (*n* = 464)**
Age (mean ± SD)	62.5 ± 1.7	63.0 ± 1.7
White race [*n* (%)]	756 (36.1)	219 (47.2)
Female [*n* (%)]	1357 (64.8)	3 (0.6)
Educational attainment [*n* (%)]		
No HS diploma	551 (26.3)	45 (9.7)
HS diploma	482 (23.0)	158 (34.1)
Some college	689 (32.9)	169 (36.4)
4-year college degree +	371 (17.7)	92 (19.8)
Diabetes [*n* (%)]	819 (39.1)	245 (52.8)
Healthy diet [*n* (%)]	1421 (67.9)	275 (59.3)
Traumatic brain injury [*n* (%)]	310 (14.8)	119 (25.6)
Probable depression [*n* (%)]	378 (18.1)	110 (23.7)
Illicit drug use (ever) [*n* (%)]	310 (14.8)	119 (25.6)
Benzodiazepine use (current) [*n* (%)]	22 (1.1)	4 (0.9)
STEM occupational history [*n* (%)]	159 (7.6)	30 (6.5)
Sedentary minutes/day (mean ± SD)	404.7 ± 211.7	472.11 ± 244.37
Word recall test (words forgotten) (mean ± SD)	1.3 ± 1.8	1.9 ± 1.5
Animal fluency test (mean ± SD)	17.1 ± 5.7	18.4 ± 5.0
Digit symbol test (mean ± SD)	48.3 ± 16.6	49.6 ± 13.01

Results of multiple linear regression models for the relationship between STEM occupational history, veteran status, and cognitive function are shown in Table S2. After controlling for relevant covariates, the interaction term for veteran status and STEM occupational history was significant (b = 2.14, $P = 0.03$) in the model using the animal fluency test as the measure of cognitive function. This interaction term was not significant in the other two models. *HS* high school, *STEM* science, technology, engineering, and mathematics

Table S2 Multiple linear regression results for the relationship between STEM occupational history, veteran status, and cognitive function test scores (*n* = 2557) [b (SE)]

Variable	Word recall test (words forgotten)	Animal fluency test	Digit symbol test
Age	0.03 (1.20)	0.03 (0.06)	-1.16 (0.15) **
White race	0.01 (0.08)	1.41 (0.25) **	7.19 (0.15) **
Female	0.04 (0.07)	0.83 (0.26) **	9.31 (0.62) **
Educational attainment			
No HS diploma	Ref	Ref	Ref
HS diploma	1.03 (0.10) **	0.15 (0.33)	4.45 (0.80) **
Some college	0.20 (0.10) *	2.45 (0.32) **	11.71 (0.77) **
4-year college degree +	0.81 (0.10) **	5.57 (0.35) **	14.22 (0.84) **
Diabetes	0.41 (0.07) **	-0.24 (0.24)	-2.65 (0.58) **
Healthy diet	0.05 (0.07)	-0.50 (0.25) *	1.91 (0.60) **
Sedentary minutes/day	-0.01 (0.01) **	0.01 (0.01) *	0.01 (0.01)
Traumatic brain injury	0.22 (0.07) **	-0.33 (0.25)	0.14 (0.61)
Probable depression	0.30 (0.08) **	-0.99 (0.28) **	-4.03 (0.68) **
Illicit drug use (ever)	0.18 (0.09)	1.04 (0.31) **	2.23 (0.77) **
Benzodiazepine use (current)	0.11 (0.25)	1.27 (0.84)	3.73 (2.03)
STEM occupation	0.07 (0.11)	-0.38 (0.37)	2.52 (0.90) **
Veteran	0.51 (0.10) **	-0.01 (0.34)	5.26 (0.83) **
STEM × veteran	-0.11 (0.30)	2.14 (1.00) *	-3.71 (2.42)

Because there were only three women veterans in the study sample, we also estimated separate multiple linear regression models for the male veteran sub-population. Results showed that male veterans with a STEM occupation had better cognitive function scores than those without a STEM occupational history, as measured by the animal fluency test (b = 3.2, $P = 0.001$) and the word recall test (b = -0.8, $P = 0.01$), but not the digit symbol test.

*$P < 0.05$, **$P < 0.01$. *Ref* reference category for the dummy variable, *b* adjusted beta coefficient, *SE* standard error, *HS* high school, *STEM* science, technology, engineering, and mathematics.

Table S3 Multiple linear regression results for the relationship between STEM occupational history and cognitive function test scores in military veterans who reported male sex (n = 461) [b (SE)]

Variable	Word recall test (words forgotten)	Animal fluency test	Digit symbol test
Age	-0.1 (0.0)	-50.0 (10.7) **	-2.0 (0.4) **
White race	1.1 (0.2) **	-0.5 (0.5)	5.7 (1.2) **
Educational attainment			
No HS diploma	Ref	Ref	Ref
HS diploma	2.5 (0.2) **	-4.1 (0.7) **	0.9 (1.8)
Some college	0.8 (0.2) **	1.4 (0.7) *	7.5 (1.6) **
4-year college degree +	1.4 (0.2) **	3.0 (0.7) **	8.3 (1.7) **
Diabetes	0.8 (0.1) **	-0.5 (0.5)	0.7 (1.2)
Healthy diet	0.1 (0.1)	-2.3 (0.5) **	-3.9 (1.2) **
Sedentary minutes/day	-0.0 (0.0) **	0.0 (0.0) **	0.0 (0.0)
Traumatic brain injury	0.7 (0.1) **	-1.4 (0.5) **	-3.4 (1.1) **
Probable depression	-0.2 (0.2)	1.22 (0.6) *	-10.1 (1.4) **
Illicit drug use (ever)	0.5 (0.2) **	-3.6 (0.6) **	-3.0 (1.4) *
Benzodiazepine use (current)	-0.1 (0.5)	2.0 (1.9)	-0.1 (4.3)
STEM occupation	-0.8 (0.3) *	3.2 (0.9) **	2.2 (2.3)

*$P < 0.05$, **$P < 0.01$. *Ref* reference category for the dummy variable, *b* adjusted beta coefficient, *SE* standard error, *HS* high school, *STEM* science, technology, engineering, and mathematics
